# Advancements in Ti_3_C_2_ MXene-Integrated Various Metal Hydrides for Hydrogen Energy Storage: A Review

**DOI:** 10.3390/nano15090673

**Published:** 2025-04-28

**Authors:** Adem Sreedhar, Jin-Seo Noh

**Affiliations:** Department of Physics, Gachon University, 1342 Seongnam-daero, Sujeong-gu, Gyeonggi-do, Seongnam-si 461-701, Republic of Korea; ademgu@gachon.ac.kr

**Keywords:** 2D Ti_3_C_2_ MXene, metal hydrides, H_2_ storage, dehydrogenation, hydrogenation

## Abstract

The current world is increasingly focusing on renewable energy sources with strong emphasis on the economically viable use of renewable energy to reduce carbon emissions and safeguard human health. Solid-state hydrogen (H_2_) storage materials offer a higher density compared to traditional gaseous and liquid storage methods. In this context, this review evaluates recent advancements in binary, ternary, and complex metal hydrides integrated with 2D Ti_3_C_2_ MXene for enhancing H_2_ storage performance. This perspective highlights the progress made in H_2_ storage through the development of active sites, created by interactions between multilayers, few-layers, and internal edge sites of Ti_3_C_2_ MXene with metal hydrides. Specifically, the selective incorporation of Ti_3_C_2_ MXene content has significantly contributed to improvements in the H_2_ storage performance of various metal hydrides. Key benefits include low operating temperatures and enhanced H_2_ storage capacity observed in Ti_3_C_2_ MXene/metal hydride composites. The versatility of titanium multiple valence states (Ti^0^, Ti^2+^, Ti^3+^, and Ti^4+^) and Ti-C bonding in Ti_3_C_2_ plays a crucial role in optimizing the H_2_ absorption and desorption processes. Based on these promising developments, we emphasize the potential of solid-state Ti_3_C_2_ MXene interfaces with various metal hydrides for fuel cell applications. Overall, 2D Ti_3_C_2_ MXenes represent a significant advancement in realizing efficient H_2_ storage. Finally, we discuss the challenges and future directions for advancing 2D Ti_3_C_2_ MXenes toward commercial-scale H_2_ storage solutions.

## 1. Introduction

To meet the target of carbon neutrality and navigate the sustainable world, researchers have continuously focused on minimizing carbon emissions. Here, the world is heading towards the development of efficient batteries and fuel cells, which deliver convenient electrical energy to divert the usage of depleting fossil fuels. Therefore, H_2_ has been considered as a promising renewable and environmentally friendly chemical fuel alternative to current fossil fuels. The major advantage of H_2_ and oxygen combination in fuel cells is water vapor (by-product) generation at zero carbon emission, which suppresses the prospect of toxic nitrogen oxides ejection into the environment [1]. This process observes electrical energy generation for its formidable utilization in moving vehicles, industries, and household purposes [2]. Due to gaining attention in the technological revolutions, H_2_ fuel vehicles are progressively expanding worldwide to achieve eco-friendly nature [3]. Furthermore, this significance lies in their potential to suppress the direct emission of greenhouse gases into the atmosphere and noise pollution. In this manner, the global demand and target of H_2_ energy production are set to 80 EJ by 2050 [4]. Thus, the scaling up of H_2_ fuel vehicles aims to increase, which necessitates controlling global warming. Majorly, H_2_ renewable energy systems can be utilized in transportation and industries, which contribute to sustainable decarbonization processes. Thus, facile H_2_ fuel storage systems and materials aligned with increasing demand. To run the H_2_ fuel vehicles, one crucial aspect of H_2_ storage material is necessary. It should be noted that H_2_ is used as a potential energy carrier in fuel cells with high energy density [5]. This feature ensures effective electrical energy generation in fuel cells. To minimize the level of H_2_ gas volume (high density) at room temperature and pressure, the following convention methods have been implemented for many years. Firstly, H_2_ has been stored in physical-based cylinders and liquid forms under high pressure and low temperatures, respectively [6]. This process is crucial for compressing the H_2_ gas and maintaining high density. In addition to the above techniques, H_2_ storage through material-based sorption (physisorption and chemisorption) offers a higher density compared to gaseous and liquid storage forms. Two different types of materials explain the physical (porous materials) and chemical (metal hydrides) absorption processes. In detail, chemisorption refers to the dissociation of H_2_ molecules into individual hydrogen atoms, which form a chemical bond with the material. This results in a higher volumetric density for H_2_ storage in metal hydrides [7]. In contrast, physisorption involves the absorption of H_2_ molecules onto a material surface without any chemical bonding, typically occurring at lower temperatures, while chemisorption is generally observed at higher temperatures. Metal hydrides are prime examples, which offer superior volumetric density compared to other storage methods. Notably, both physisorption and chemisorption processes do not require catalysts as H_2_ molecules are directly absorbed and desorbed from the material surface. The absorption and desorption efficiency strongly depends on the bonding energy of H_2_ atoms and the material’s thermal stability, and both are critical factors in optimizing H_2_ storage performance. This feature highlights the potential use of H_2_ storage materials in fuel cell vehicles. Interestingly, more H_2_ can be stored in small vessels using solid H_2_ storage materials than in liquid compressed cylinders. One promising method for H_2_ production is through photocatalytic and photoelectrochemical water splitting techniques [8,9]. As mentioned earlier, the generation of water vapor as a by-product in the fuel cells can be harnessed to produce H_2_ fuel and O_2_ through photocatalytic processes. This H_2_ fuel can be stored in H_2_ storage materials. Interestingly, the integration of the photocatalytic effect (H_2_ fuel generation), H_2_ storage, and H_2_ combustion (energy generation with water vapor generation as by-product) offers a potential path toward a sustainable clean energy future [10]. Furthermore, the cost of manufacturing H_2_ fuel cells is lower than that of current battery technologies [11]. Additionally, H_2_ energy content (120 MJ/kg) is three times higher than that of gasoline (44 MJ/kg) [12].

Conventional H_2_ storage systems require low density and low temperature conditions, such as the extremely low temperature of −252.87 °C. Metal hydrides, carbonaceous materials, and metal–organic frameworks (MOFs) are promising candidates for solid H_2_ storage. Among these, metal hydroxides have demonstrated superior H_2_ storage capacity compared to liquid and pressurized conditions. This can be attributed to the chemisorption process, which allows H_2_ to be stored within solid materials under moderate temperature and pressure. During absorption and desorption cycles, the H_2_ atoms in the metal hydrides induce rearrangements in the crystal structure, leading to volume expansion [13]. Notably, the highest H_2_ density has been observed in the following order: chemisorption > physisorption > liquid form > pressure form [14]. Metal hydrides offer a safe approach for achieving high H_2_ storage densities, efficient desorption at low temperatures, and enhanced cyclic stability. Several metal hydrides and mixed metal hydrides, such as MgH_2_, LiBH_4_, NaMgH_3_, NaAlH_4_, NaBH_4_, and Mg(BH_4_)_2_ have been extensively investigated. Recent research on MgH_2_ has highlighted its potential for H_2_ storage, particularly when combined with a single-atom Ni supported on TiO_2_, which achieved H_2_ absorption of 6.53 wt% in just 10 s [15]. Moreover, when MgH_2_ was combined with LiBH_4_, Ding et al. [16] demonstrated a reversible H_2_ storage capacity of 5.0 wt% at temperatures below 265 °C, outperforming other MgH_2_ + LiBH_4_ systems. Additionally, MgH_2_ combined NaAlH_4_ exhibited a H_2_ storage capacity of 7.42 wt% after 60 min [17]. The combination of MgH_2_ with NaBH_4_ showed promising rehydrogenation results, reaching 5.89 wt% in 12 h at 600 °C [18]. These studies represent significant advancements in H_2_ storage capacity through the development of complex hybrid structures. Many researchers provided the reviews on the importance of magnesium (Mg)-based materials [19], rare earth-Mg based alloys [20], which explained the role of rare earth-Mg based alloys for H_2_ storage performance. Furthermore, Li et al. [21] provided the systematic information on thermodynamics and kinetics relating to the dehydriding and hydriding process of Mg based materials. Interestingly, the combination of NbC and Nb_4_C_3_ achieved the superior H_2_ storage performance of the Li–Mg–B–H composite [22]. In another study, Lu et al. [23] also studied the H_2_ storage performance of Li–Mg–B–H composite (2LiBH_4_ + MgH_2_) by the addition of Nb_2_C. The role of fluorine-functionalized intercalation in graphene was explained for H_2_ storage of LiBH_4_ by adjusting the interlayer space [24]. 

The H_2_ storage capabilities of metal hydrides mentioned above can be significantly enhanced by incorporating a novel Ti metal, particularly in the form of two-dimensional (2D) Ti_3_C_2_ MXene. A linear improvement in H_2_ uptakes has been observed as the surface area of the resultant material increases [25]. The 2D Ti_3_C_2_ MXene interacts, notably, with CO_2_ and N_2_ during photocatalytic and electrocatalytic reduction processes [26]. This highlights the critical role of Ti_3_C_2_ MXene, with its high surface area and layer structure, in facilitating the H_2_ storage process. The theoretical studies using density functional theory indicated that the accessibility of Ti_2_C MXene can achieve a H_2_ storage capacity of 8.5 wt% [27]. Experimental results confirmed that multilayer Ti_2_C MXene exhibited a H_2_ uptake of 8.8 wt% at room temperature [28]. Furthermore, Ti_3_C_2_ has demonstrated its potential in promoting a sustainable clean energy environment [29]. In addition to Ti_3_C_2_, V_2_C also proved potential H_2_ storage performance [30]. Previously, the role of MXenes towards H_2_ storage of various metal hydrides has been successfully reported [31,32]. Therefore, the 2D Ti_3_C_2_ MXene community offers distinct advantages for developing an efficient H_2_ storage system.

## 2. Importance of Ti_3_C_2_ MXene for H_2_ Storage

**Figure 1:**
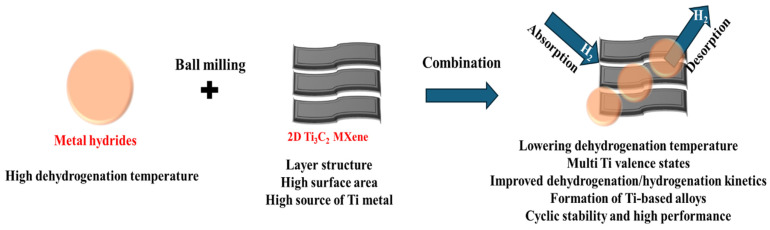
Schematic illustration of metal hydrides mixed with 2D Ti_3_C_2_ MXene for improvement in H_2_ storage performance.

The hydrogen storage (absorption) process in MgH_2_ can be enhanced by considering the layered structure and titanium-rich characteristics of Ti_3_C_2_. Specifically, titanium (Ti) metal forms through the strategic interaction between MgH_2_ and Ti_3_C_2_, which act as promising materials for H_2_ absorption. It should be noted that the Ti functioned [2,2,2] paracyclophane explained the H_2_ storage performance under the strategic transition from chemisorption to physisorption [33]. Additionally, the distinct 2D layered structure and high surface area of Ti_3_C_2_ provide more active sites for further improving hydrogen storage capabilities. Recently, pure 2D Ti_3_C_2_T_x_ MXene achieved an experimental hydrogen storage capacity of 10.47 wt% under 25 bar and 77 K for the first time. Theoretical studies on H_2_ storage were explained by the various MXene bilayers [34]. Figure 1 shows the significance of the layered 2D Ti_3_C_2_ MXene structure in enhancing the H_2_ storage performance of different metal hydrides. Research on Ti_3_C_2_ MXene-based metal hydrides for H_2_ storage is still in its early stages. Meanwhile, there are few reviews addressing the potentiality of MXenes for H_2_ storage in fuel cell applications [4,35,36]. However, there is a lack of detailed focus on the evaluation of Ti_3_C_2_ MXene-based metal hydrides, specifically for H_2_ storage. This review aims to explore the potential role of 2D Ti_3_C_2_ MXene in enhancing the H_2_ storage performance of various metal hydrides.

**Figure 2:**
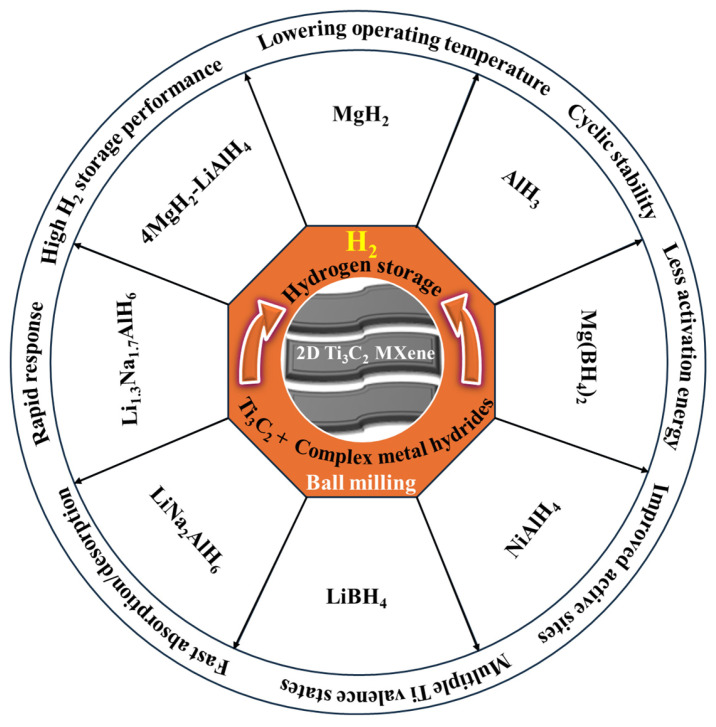
Potentiality of various metal hydrides mixed 2D Ti_3_C_2_ MXenes for H_2_ storage performance.

Based on the above key insights, we have investigated the integration of various metal hydrides with 2D Ti_3_C_2_ MXene to enhance H_2_ storage performance at lower operating temperatures. Figure 2 illustrates a schematic representation of ball milled metal hydrides combined with 2D Ti_3_C_2_ MXene, aimed at future fuel cell applications. This combination is designed to improve the dehydrogenation and hydrogenation kinetics at lower operating temperatures.

## 3. Role of Ti_3_C_2_ MXene/Metal Hydride Interface for H_2_ Storage

Due to the key structural and surface termination features of Ti_3_C_2_ MXene, the combination of Ti_3_C_2_ MXene and metal hydrides strengthens the overall H_2_ storage capacity. Specifically, the Ti_3_C_2_ MXene has the ability to reduce the desorption temperature of metal hydrides as a potential catalyst by the creation of Ti metal and various Ti valance states (Ti^0^, Ti^2+^, Ti^3+^, and Ti^4+^). Moreover, Ti_3_C_2_ MXene stabilizes the H_2_ desorption of metal hydrides and increases the cyclic stability of the resultant composite. It should be noted that the diffusion of hydrogen molecules can be significantly improved in metal hydrides by the addition of Ti_3_C_2_ MXene to improve the absorption/desorption kinetics. Thus, the Ti_3_C_2_ MXene/metal hydride combination results in improved H_2_ storage performance at lower temperatures, compared with pure metal hydrides, especially for its applicability in automobile industry.

## 4. Ti_3_C_2_ MXene-Based H_2_ Storage Materials

As we explained before, metal hydrides and mixed metal hydrides play a crucial role in the H_2_ storage process. The integration of layer-structured Ti_3_C_2_ MXene with metal hydrides has further attracted the efficiency of H_2_ storage. Researchers have continuously focused on the unique physical and chemical properties of Ti_3_C_2_ MXene to better understand its role in hydrogen storage and its potential applications in fuel cell vehicles. Given the significance of various metal hydride interaction networks with 2D Ti_3_C_2_ MXene, we have conducted a thorough evaluation of the efficiency of the H_2_ absorption and desorption processes.

### 4.1. Binary Metal Hydrides

#### 4.1.1. MgH_2_

The ball milling technique has been successfully used to create a synergy between MgH_2_ and few- or multi-layer Ti_3_C_2_ MXene, leading to significant improvements in the H_2_ absorption and desorption processes compared to pure MgH_2_. Additionally, the presence of Ti in various oxidation states within Ti_3_C_2_ MXene helps to lower the operating temperatures in contrast to pure MgH_2_. Sharp H_2_ release and absorption observed through the formation of MgH_2_ involved Ti_3_C_2_ MXene. The potential role of Ti_3_C_2_ in optimizing H_2_ storage performance of MgH_2_ is explained below.

The potential of Ti metal in Ti_3_C_2_ for enhancing the rapid dehydrogenation and hydrogenation process at various operating temperatures was investigated [37]. In this study, a selective combination of ball milled Ti_3_C_2_ (5 wt%) with MgH_2_ (MgH_2_-5 wt% Ti_3_C_2_) altered both the operating temperature and dehydrogenation process. During ball milling, metallic Ti was generated in situ, which significantly promoted the dissociation and recombination of molecular H_2_ on the MgH_2_-5 wt% Ti_3_C_2_ composite. Specifically, Ti_3_C_2_ contents (0, 1, 3, 5, and 7 wt%) were varied to explore their effects on the non-isothermal and isothermal dehydrogenation/hydrogenation processes. This optimized sample reduced the dehydrogenation operating temperature from 278 °C (pure MgH_2_) to 185 °C. Under isothermal conditions at 300 °C, the H_2_ desorption reached approximately 6.2 wt% within 1 min. Furthermore, with the dehydrogenated samples exposed to 50 bar hydrogen pressure, a H_2_ uptake of 6.1 wt% was achieved in just 30 s at 150 °C under isothermal conditions. Notably, H_2_ absorption started at room temperature and 5.5 wt% uptake was achieved at 100 °C under non-isothermal conditions. Overall, the combination of MgH_2_ and Ti-rich Ti_3_C_2_ (without Ti-C bonding) was highly effective in enhancing the cyclic stability of hydrogen storage, achieving 95% retention capacity after 10 cycles.

In another study, Gao et al. [38] explained the reduction in the operating temperature of the dehydrogenation process through the synergistic combination of carbon (C)-supported 5 wt% Ti_3_C_2_/TiO_2_-C and MgH_2_. In this case, Ti_3_C_2_ was partially oxidized to TiO_2_ and C under CO_2_ at 600 °C. The presence of multiple valance states of Ti (Ti^0^, Ti^2+^ Ti^3+^, and Ti^4+^) in the Ti_3_C_2_/TiO_2_-C composite facilitated rapid charge transfer, which enabled the conversion between Mg^2+^ and Mg or H^−^ and H. This effect significantly enhanced the H_2_ absorption and desorption process. After 5 wt% Ti_3_C_2_/TiO_2_-C was ball milled with MgH_2_ in Ar atmosphere, the composite released 5 wt% H_2_ in 1700 s at 250 °C under 0.05 MPa pressure. The rate constant observed was 0.258 wt% min^−1^ for MgH_2_-5 wt% Ti_3_C_2_/TiO_2_ (A)-C, which was 1.48 and 9.6 times higher than that of MgH_2_-5 wt% Ti_3_C_2_ and MgH_2_-5 wt% Ti_3_C_2_(A)-C, respectively. Additionally, these dehydrogenated samples absorbed 4 wt% of H_2_ in 800 s at 125 °C under isothermal conditions, outperforming MgH_2_-5 wt% Ti_3_C_2_ (3 wt%) and MgH_2_-5 wt% TiO_2_ (A)-C (2.65 wt%). This study clearly demonstrated that the Ti metal preset in Ti_3_C_2_ plays a crucial role in triggering H_2_ absorption and desorption at lower operating temperatures.

**Figure 3:**
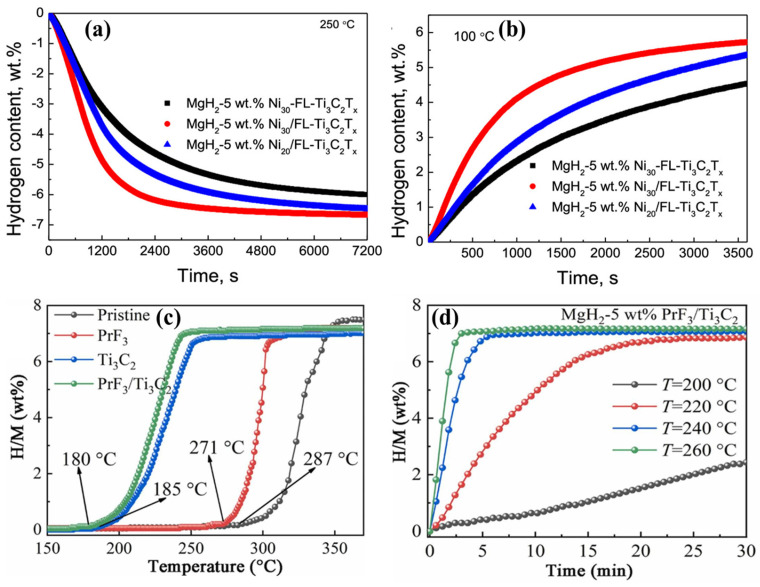
(**a**) Isothermal dehydrogenation behavior of 5 wt% Ni_30_/FL-Ti_3_C_2_T_x_ doped MgH_2_ at 250 °C, (**b**) isothermal hydrogenation of 5 wt% Ni_30_/FL-Ti_3_C_2_T_x_ doped MgH_2_ at 100 °C (reprinted from Ref. [39] copyright 2020, with permission from ACS Publication), (**c**) non-isothermal dehydrogenation behavior of PrF_3_/Ti_3_C_2_ interfaced MgH_2_ compared to pristine, PrF_3_, and Ti_3_C_2_, and (**d**) isothermal H_2_ desorption at 260 °C (reprinted from Ref. [40] copyright 2022, with permission from Elsevier).

In addition to the conventional multilayer Ti_3_C_2_T_x_ network, few-layer (FL) Ti_3_C_2_T_x_ MXene supported MgH_2_ has been explored for the first time in the H_2_ desorption/absorption process without altering its surface features [39]. In this study, an electrostatic self-assembly reduction process was used for in situ growth of 20–140 nm nano-Ni particles (at 20, 30, and 40 wt%) within FL Ti_3_C_2_T_x_ MXene (1.42 nm interlayer distance), which was then ball milled with MgH_2_. As in previous studies [38], the potential contributions of Ti^0^, Ti^2+^, Ti^3+^, and Ti^4+^ valence states were discussed in relation to H_2_ storage performance. The 5 wt% Ni_30_/FL-Ti_3_C_2_T_x_ doped MgH_2_ demonstrated dehydrogenation of 5.83 wt% in 1800 s 250 °C under 0.005 MPa (isothermal dehydrogenation) as shown in Figure 3a. The same sample also exhibited improved isothermal hydrogenation, reaching 5 wt% in 1700 s under 3.0 MPa H_2_ pressure at 100 °C (Figure 3b). Furthermore, stable cyclic hydrogenation and dehydrogenation were observed over 10 cycles at 100 °C with minimal attenuation of approximately 0.06 wt% per cycle. A new phase of MgO was formed during the testing process, which may explain the decrease in cyclic stability.

Similarly, Ni nanoparticles supported on monolayer Ti_3_C_2_ MXene (Ti-MX) were synthesized using a self-assembly technique and combined with MgH_2_ through ball milling (MgH_2_ + Ni@Ti-MX) to facilitate H_2_ absorption and release at lower operating temperatures [41]. During the dehydrogenation of MgH_2_ in the presence of Ni along with Ti, the formation of Mg_2_Ni was observed. The interfaces between Mg/Mg_2_Ni, Mg/TiO_2_, Mg/Ti, and Mg/C were identified, playing a key role in triggering the H_2_ absorption process of MgH_2_. The H_2_ absorption of dehydrogenated MgH_2_ + Ni@Ti-MX was observed at 125 °C under 3 MPa, releasing 5.4% H_2_ in 25 s (4.0 wt% at 75 °C in 60 min). The rapid absorption process (25 s) was significantly faster than the previously studied Ni_30_/FL-Ti_3_C_2_T_x_ (1700 s) [39]. Notably, room temperature H_2_ absorption (4.0 wt% in 5 h) was also observed. The activation energy of 56 ± 4 KJ/mol H_2_ was achieved, which was higher than other MXene composites, such as MgH_2_−5 wt % Nb_4_C_3_T_x_ (27.8 kJ/mol H_2_) [42] and MgH_2_−10 wt % Ni/CMK-3 (37 kJ/mol H_2_) [43]. The isothermal dehydrogenation process reached 5.2 wt% in 15 min at 250 °C and 5.0 wt% H_2_ at 300 °C in 200 s. The 2D Ti_3_C_2_ MXene facilitated H_2_ diffusion during the absorption/release cycle, demonstrating excellent cyclic stability over 10 cycles at 275 °C. Overall, the presence of Ni nanoparticles further enhanced H_2_ storage performance in the MgH_2_ + Ni@Ti-MX composite.

Additionally, Ti_3_C_2_ MXene-derived K_2_Ti_6_O_13_ (Hamamelis-like structure) in a KOH + H_2_O_2_ environment enhanced the H_2_ storage performance of MgH_2_ [44]. The H_2_ storage behavior of MgH_2_-K_2_Ti_6_O_13_ was studied at 0, 3, 5, and 10 wt% concentrations of K_2_Ti_6_O_13_. According to the temperature-programmed desorption (TPD) results, the ball milled MgH_2_-K_2_Ti_6_O_13_ was subjected to 1.5 MPa of H_2_, and showed a reduction in the onset and termination temperature to 175 °C and 220 °C, respectively, with 5 wt% of K_2_Ti_6_O_13_ compared to pure MgH_2_ (287 °C–onset). The isothermal dehydrogenation performance exhibited a 6.7 wt% H_2_ release at 280 °C in 3 min, which was 101.5 times higher than that of pure MgH_2_. Subsequently, the dehydrogenated sample absorbed approximately 6.5 wt% H_2_ in 30 s at 200 °C at 2.2 MPa (4.8 wt% at 100 °C in 5 min). The cyclic stability of dehydrogenation (280 °C) and hydrogenation (200 °C) under 2.2 MPa remained stable after 10 cycles. The charge (electron) transfer between Ti and Ti^2+^ accelerated the diffusion of H_2_. Moreover, KMgH_3_ formation during ball milling process further accelerated the H_2_ diffusion during both absorption and desorption processes.

In another study, the combination of dual V_2_C and Ti_3_C_2_ MXenes enhanced the H_2_ storage capacity of MgH_2_ at 10 wt % of 2V_2_C/Ti_3_C_2_ [45]. After successful ball milling, H_2_ desorption and H_2_ absorption processes, V-C and Ti-C bonding were observed, highlighting the critical role V_2_C and Ti_3_C_2_ as catalysts in the H_2_ storage process. The uniform distribution of Mg, Ti, and V elements in the MgH_2_-V_2_C/Ti_3_C_2_ composite promoted the H_2_ absorption activity of MgH_2_. Furthermore, the presence of various Ti valance states contributed to improving the H_2_ absorption capacity of MgH_2_. The addition of 2V_2_C/Ti_3_C_2_ reduced the starting desorption temperature of MgH_2_ from 320 °C to 180 °C. The non-isothermal studies showed a 6.3 wt% H_2_ release at 250 °C. The non-isothermal H_2_ desorption performance of MgH_2_-2V_2_C/Ti_3_C_2_ resulted in 5.1 wt% H_2_ release within 60 min at 225 °C and 5.8 wt% in just 2 min at 300 °C. Accordingly, MgH_2_-2V_2_C/Ti_3_C_2_ demonstrated 5.1 wt% of H_2_ absorption in 20 s at 40 °C under isothermal conditions. This configuration maintained 10 cycles of desorption stability at 300 °C with 6.3 wt% H_2_ release. The activation energy of H_2_ desorption in MgH_2_-2V_2_C/Ti_3_C_2_ was 79.4 kJ mol^−1^ H_2_. Overall, the dual 2D MXene configuration in MgH_2_ reduced H_2_ absorption time to 20 s and the operating temperature to 40 °C with 5.1 wt% absorption.

Wu et al. [46] highlighted the significance of multilayer Ti_3_C_2_ in enhancing the H_2_ storage of MgH_2_ potentiality through the involvement of Ti metal. The Ti metal, formed in situ during the ball milling process of Ti_3_C_2_ and MgH_2_, played a crucial role in improving H_2_ absorption and desorption. In this study, Ti_3_C_2_ at 6 wt% was optimized in MgH_2_ (MgH_2_-6 wt.% ML-Ti_3_C_2_). During the non-isothermal process, the initial dehydrogenation temperature was 142 °C with a 6.56 wt% H_2_ release. Following the dehydrogenation, the non-isothermal H_2_ absorption efficiency of 6.3 wt% was observed at room temperature (30 °C) compared to MgH_2_ (70 °C). Under isothermal conditions, the H_2_ storage and desorption capacities were about 6.47 and 6.45 wt% at 150 and 240 °C, respectively. The activation energy for the dehydrogenation process of MgH_2_-6 wt.% ML-Ti_3_C_2_ was about 99.11 kJ/mol, lower than that of MgH_2_ (153.09 kJ/mol). This suggests that the addition of Ti_3_C_2_ facilitates faster H_2_ release in MgH_2_. During the dehydrogenation processes, MgH_2_ was converted into Mg and MgO. Whereas Mg was converted back into MgH_2_ during the rehydrogenation process.

In addition to the nano-Ni particles [39] and Ni nanoparticles [41], Gao et al. [47] investigated the interaction of Ni particles (<50 nm) with Ti_3_C_2_ (Ni/Ti_3_C_2_-WE) to enhance the H_2_ storage capacity of MgH_2_. Specifically, ball milled MgH_2_ with 5 wt% Ni/Ti_3_C_2_ was optimized to study the hydrogenation/dehydrogenation processes. The absorption and desorption kinetics of MgH_2_ were significantly influenced by the excellent interface between Ni and Ti_3_C_2_. The presence of multiple Ti valence states (Ti^0^, Ti^2+^ Ti^3+^, and Ti^4+^) again proved electron transfer, which enhanced the catalytic activity of Ni/Ti_3_C_2_-WE. Non-isothermal studies showed that, at 240 °C, a dehydrogenation of 3.02 wt% was achieved. Under isothermal conditions, the dehydrogenated MgH_2_-5 wt% Ni/Ti_3_C_2_ absorbed 4.59 wt% H_2_ in 1200 s at 100 °C (MgH_2_-1.67 wt% at 200 °C). Remarkably, the system also demonstrated impressive low temperature (50 °C) H_2_ absorption, with 4.51 wt% in 5.5 h, meeting the U.S. Department of Energy (DOE) criteria for light-duty vehicle applications. The desorption of H_2_ reached 5.87 wt% and 6.73 wt% in 2400 s at 250 °C and 300 °C, respectively. Additionally, the MgH_2_-5 wt% Ni/Ti_3_C_2_ demonstrated stable absorption/desorption cycles over 10 cycles at 275 °C. Overall, the dissociation and recombination of H_2_ were facilitated by the Ti metal formation and mitigation of surface passivation of MgH_2_ in the MgH_2_-5 wt% Ni/Ti_3_C_2_.

In another study, the uniform distribution of praseodymium fluoride (PrF_3_) nanoparticles on Ti_3_C_2_ (PrF_3_/Ti_3_C_2_) enhanced the H_2_ storage capacity of MgH_2_ [40]. The valence states of Ti (Ti^2+^, Ti^3+^, and Ti^4+^) created the active sites for charge (electron) transfer between Mg^2+^ and H^−^, facilitating a significant dehydrogenation process. Specifically, ball milling of PrF_3_/Ti_3_C_2_ with MgH_2_ resulted in the formation of Ti metal and various Ti valence states (Ti^0^, Ti^2+^, Ti^3+^, and Ti^4+^), which improved the dissociation and recombination of H_2_ molecules. Additionally, enhanced charge transfer between Ti^3+^ and Ti^2+^ was observed during both the hydrogenation and dehydrogenation processes. Under non-isothermal conditions, the hydrogen desorption of 7.2 wt% was achieved at 230 °C, as shown in Figure 3c. Isothermal studies on MgH_2_-5 wt% PrF_3_/Ti_3_C_2_ demonstrated 7.0 wt% H_2_ desorption in 3 min at 260 °C and H_2_ absorption of 6.16 wt% at 150 °C within 10 min, as shown in Figure 3d. The non-isothermal dehydrogenation achieved a 7.2 wt% capacity starting at 180 °C. The dehydrogenation activation energy for MgH_2_-5 wt% PrF_3_/Ti_3_C_2_ was 78.11 kJ mol^−1^, which was lower than that of pure MgH_2_ (117.98 kJ mol^−1^). Furthermore, the composite retained 92.5% of its capacitance after 10 cycles of dehydrogenation.

**Figure 4:**
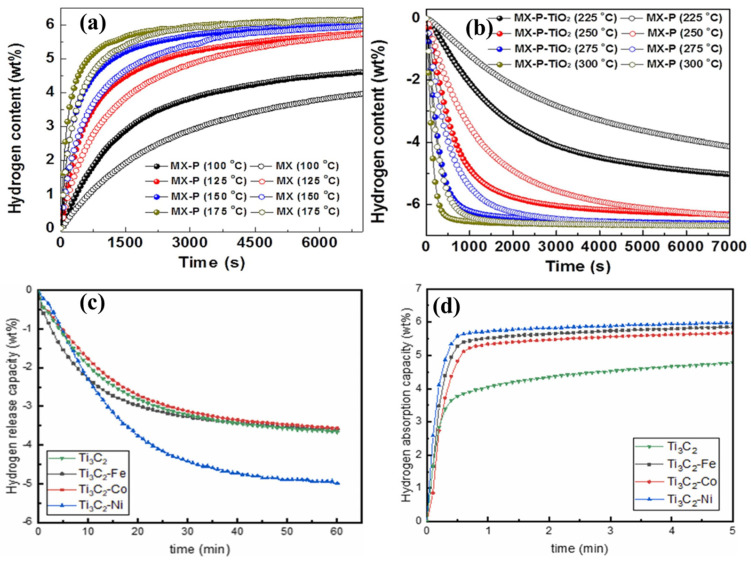
(**a**) Isothermal hydrogenation behavior of porous Ti_3_C_2_ MXene (MX) mixed MgH_2_ at 100 °C, (**b**) isothermal desorption behavior of MgH_2_-5 wt% MX-P-TiO_2_ (reprinted from Ref. [48] copyright 2023, with permission from Elsevier), (**c**) isothermal desorption behavior of pure Ti_3_C_2_, and TiCrV/Ti_3_C_2_-(Fe, Co, and Ni) at 523 K, and (**d**) isothermal H_2_ absorption of pure Ti_3_C_2_, and TiCrV/Ti_3_C_2_-(Fe, Co, and Ni), at 453 K (reprinted from Ref. [49] copyright 2024, with permission from Elsevier).

The formation of internal edge planes on porous Ti_3_C_2_ MXene (MX-P) was shown to enhance the H_2_ storage in MgH_2_ by increasing the specific surface area and creating internal metallic Ti active sites [48]. The exposed internal Ti edge sites raised the Ti content in MgH_2_-5 wt% MX-P to 33.3% compared to pure Ti_3_C_2_ MXene (20.3%) in MgH_2_-5 wt% MX. In situ oxidation and etching processes created small holes on the surface of MX-P-TiO_2_, which partially converted the Ti active sites into TiO_2_. By adjusting the etching time, the number of holes and internal edge sites could be significantly increased. The isothermal hydrogenation process revealed a 2.55 wt% H_2_ uptake at 100 °C in 1200 s (Figure 4a). Through these beneficial surface features, both MgH_2_-5 wt% MX-P-TiO_2_ and MgH_2_-5 wt% MX-P exhibited H_2_ storage capacities of 6.6 and 6.5 wt%, respectively, after 10 cycles at 275 °C. The isothermal desorption behavior of MgH_2_-5 wt% MX-P-TiO_2_ at various temperatures is shown in Figure 4b. Additionally, the absorption activation energy for MgH_2_-5 wt% MX-P-TiO_2_ (33.95 kJ mol^−1^) was lower than that of MgH_2_-5 wt% MX-P (36.22 kJ mol^−1^) and MgH_2_-5 wt% MX (43.10 kJ mol^−1^). Overall, the creation of internal edge planes in Ti_3_C_2_ MXene significantly increased the Ti active sites for H_2_ absorption in MgH_2_-5 wt% MX-P-TiO_2_.

Furthermore, the synergistic interaction between MnO_2_ nanoparticles and 2D Ti_3_C_2_ significantly influenced the H_2_ storage performance of MgH_2_ in the MgH_2_ + Ti_3_C_2_@MnO_2_ composite at room temperature [50]. In this system, Mn, MnO_2_, MnO, TiO_2_, and Ti_3_C_2_ act as common catalysts during the hydrogenation and dehydrogenation process. During hydrogenation, Mg was converted into MgH_2_. The formation of multiple interfaces, including Ti_3_C_2_/MgH_2_, TiO_2_/MgH_2_, MnO_2_/MgH_2_, MnO/MgH_2_, and Mn/MgH_2_ created numerous channels for hydrogen atom diffusion, enhancing the H_2_ absorption and desorption processes of the Mg/MgH_2_ system. Isothermal hydrogenation achieved 4.4 wt% H_2_ absorption at 30 °C within 150 s and 5.13 wt% at 75 °C in 400 s (MgH_2_-0.28 wt% at 150 °C in 400 s). Dehydrogenation reached 6.4 wt% at 275 °C within 484 s. Remarkably, these interfaces exhibited 20-cycle stability at 275 °C with hydrogen absorption of 6.37 wt%, confirming the stability of the developed composite and its excellent hydrogen absorption/desorption performance.

In another study, a combination of Ni nanoparticles and Ti_3_C_2_ MXene enhanced the H_2_ desorption behavior of MgH_2_ [51]. Ball milling of Mg, Ni, and Ti_3_C_2_ produced Mg-xNi/Ti_3_C_2_ composites at different weight ratios (5 wt% of xNi/Ti_3_C_2_, x = 0.5, 1, 2, and 3) with 95 wt% Mg. The Ni content in Ti_3_C_2_ (Ti_3_C_2_/Ni = 1:2) and 5 wt% Ti_3_C_2_ content significantly influenced the H_2_ storage performance. At 300 °C, the Mg-2Ni/Ti_3_C_2_ composite exhibited H_2_ absorption and released values of 4.46 wt% and 3.96 wt%, respectively. The isothermal dehydrogenation of Mg-2Ni/Ti_3_C_2_ was 4.54 wt% in 5 min compared to the 3.76 wt% for pure Mg after in 70 min at 375 °C. The synergy between Ni and Ti_3_C_2_ lowered the activation energy of Mg-2Ni/Ti_3_C_2_ to about 75.0 kJ mol^−1^ (Mg-135.4 kJ mol^−1^). During hydrogenation, Mg was converted into MgH_2_ and during dehydrogenation, the system formed Mg, MgO, and Mg_2_Ni. The characteristic feature of Ti^3+^ disappeared after the dehydrogenation process, leaving Ti, Ti-C, and Ti^2+^ as the remaining phases. The metallic Ti developed during ball milling facilitated H_2_ desorption, with Ti_3_C_2_ acting as a H_2_ pump. Additionally, Ti_3_C_2_ played a crucial role in controlling the expansion of the Mg pellet, ensuing stability in the H_2_ absorption and desorption cycles.

In another study, Ti_3_C_2_ was coordinated with 3d transition metal (Fe, Co, Ni) particles to enhance the H_2_ storage performance of the MgH_2_-TiCrV composite [49]. Ball milling of Ti_3_C_2_ with Fe, Co, Ni, and Mg-TiCrV resulted in the formation of Mg-TiCrV/Ti_3_C_2_-Fe, Co, Ni composites, which reduced the layer structure and promoted the development of cluster morphology. These composites were evaluated for dehydrogenation at temperatures of 498, 523, 543, and 573 K under 0.1 MPa. At 523 K, the Mg-TiCrV/Ti_3_C_2_-Ni released the H_2_ about 4.982 wt% in 60 min, outperforming other temperatures and TiCrV/Ti_3_C_2_-Fe (3.598 wt%) and TiCrV/Ti_3_C_2_-Co (3.789 wt%), as shown in Figure 4c. The H_2_ release capability was further enhanced at 543 K, reaching 5.447 wt% for TiCrV/Ti_3_C_2_-Ni. Furthermore, TiCrV/Ti_3_C_2_-Ni exhibited a rapid isothermal H_2_ absorption of 5.72 wt% in just 1 min at 453 K, surpassing the absorption of the TiCrV/Ti_3_C_2_-Fe (5.53 wt%) and TiCrV/Ti_3_C_2_-Co (5.34 wt%) composites (Figure 4d). Based on these findings, it was concluded that Ni coordination was more effective than Fe and Co (Ni > Fe > Co) in enhancing the performance of Mg-TiCrV/Ti_3_C_2_-Fe, Co, Ni composites. Furthermore, the activation energy for H_2_ release was lower for TiCrV/Ti_3_C_2_-Ni (80.54 kJ mol^−1^) compared to TiCrV/Ti_3_C_2_-Fe (88.28 kJ mol^−1^) and TiCrV/Ti_3_C_2_-Co (88.90 kJ mol^−1^). Overall, the inclusion of 3D transition metals significantly improved the H_2_ storage performance of Mg-TiCrV composite.

The above results clearly demonstrated the role of Ti metal, its valance states (Ti^0^, Ti^2+^, Ti^3+^, and Ti^4+^), and Ti-C bonds in the 2D Ti_3_C_2_ MXene for enhancing the H_2_ storage performance of MgH_2_.

#### 4.1.2. AlH_3_

In addition to MgH_2_, AlH_3_ has been observed with high H_2_ densities of 10.1 mass% and 149 gH_2_ L^−1^ [52]. The H_2_ storge performance of AlH_3_ was observed at low temperatures, ranging from 150 to 200 °C [53]. The H_2_ volume density of AlH_3_ is double that of liquid H_2_, providing significant potential for H_2_ storage. However, AlH_3_ is highly reactive with oxygen and water, which limits its broader applicability H_2_ storage. To improve its H_2_ storage performance, effective decomposition of AlH_3_ is necessary.

In this context, the potential of H_2_ storage performance was significantly enhanced by the strategic air-ball milling of AlH_3_ and Ti_3_C_2_, which resulted in the formation of an Al_2_O_3_ layer on AlH_3_ for the first time [54]. This strategy facilitated close contact between the AlH_3_ oxide layers and Ti_3_C_2_, which effectively minimized the loss of H_2_ storage capacity. The extended air-milling process (an additional 60 min) led to a reduction in the initial decomposition temperature (61 °C) and increased H_2_ release (8.1 wt%) at 4 wt% Ti_3_C_2_. Under isothermal conditions (100 °C), the AlH_3_ + 4 wt% Ti_3_C_2_ mixture released 6.9 wt% H_2_ in 20 min and 4.9 wt% in 10 min. In comparison, milled AlH_3_ alone released 0.1 wt% and 0.3 wt% at 100 °C in 10 and 20 min, respectively. The dehydrogenation activation energy of AlH_3_ + 4 wt% Ti_3_C_2_ was 40 kJ mol^−1^ (milled AlH_3_-99 kJ mol^−1^). The process of AlH_3_ and Ti_3_C_2_ combination triggered the dehydrogenation kinetics performance. The combination of AlH_3_ and Ti_3_C_2_ accelerated the dehydrogenation kinetics, while the Al_2_O_3_ layer on AlH_3_ was protected during the air-ball milling process, preserving the catalyst. Moreover, the layer structured Ti_3_C_2_ with a high surface area and active sites created a robust connection with AlH_3_, enabling efficient H_2_ storage at low temperatures.

Overall, the 2D Ti_3_C_2_ MXenes had a significant impact on the H_2_ storage performance of MgH_2_ and AlH_3_ by reducing the operating temperature. The binary metal hydrides (MgH_2_ and AlH_3_) integrated Ti_3_C_2_ MXenes at a specific weight percentage and key factors for H_2_ storage performance are summarized in Table 1.

### 4.2. Ternary Metal Hydrides

#### 4.2.1. Mg(BH_4_)_2_

**Figure 5:**
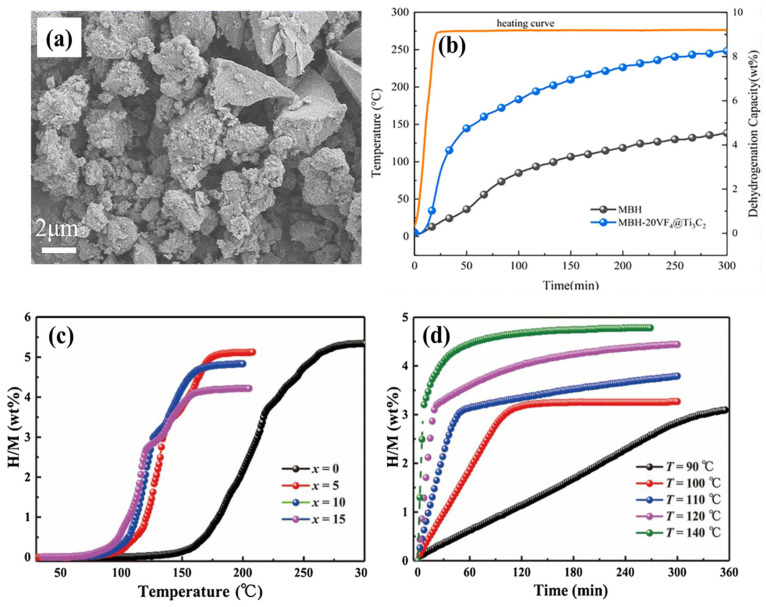
(**a**) Surface morphology of Mg(BH_4_)_2_-20VF_4_@Ti_3_C_2_, (**b**) isothermal H_2_ desorption of Mg(BH_4_)_2_-20VF_4_@Ti_3_C_2_ at 275 °C (reprinted from Ref. [55] copyright 2023, with permission from Elsevier), (**c**) non-isothermal dehydrogenation process of NaAlH_4_ + MXene/A-TiO_2_ at various weight ratio of MXene/A-TiO_2_, and (**d**) isothermal H_2_ dehydrogenation of NaAlH_4_ + 10 wt% MXene/A-TiO_2_ at 140 °C (Reprinted from Ref. [62] copyright 2018, with permission from Elsevier).

Zhang et al. [55] developed VF_4_ nanoparticles anchored on 2D Ti_3_C_2_ to evaluate the H_2_ storage performance of Mg(BH_4_)_2_. The presence of Ti_3_C_2_ MXene significantly reduced the aggregation of Mg(BH_4_)_2_ during the absorption and desorption processes. In the TPD process, VF_4_@Ti_3_C_2_ (20 wt%) demonstrated that the dehydrogenation began at 90 °C and reached a 13 wt% H_2_ release by 500 °C. The SEM image of Mg(BH_4_)_2_-20VF_4_@Ti_3_C_2_ is shown in Figure 5a. The isothermal desorption process maintained a H_2_ release of 8.2 wt% at 275 °C in 300 min for Mg(BH_4_)_2_-4.5 wt% (Figure 5b). The activation energy for the first step of dehydrogenation of Mg(BH_4_)_2_-20 VF_4_@Ti_3_C_2_ was 172.099 kJ mol^−1^, lower than that of pure Mg(BH_4_)_2_ (374.1 kJ mol^−1^). This reduction in activation energy was attributed to the destabilization of the B–H bonds in the composite. Ti_3_C_2_ remained stable during the de/hydrogenated processes, functioning as a scaffold and exhibiting synergistic behavior with VH_2.01_ and Mg(BH_4_)_2_ for enhancing the H_2_ release and uptake processes. Overall, the formation of metallic Ti and VH_2.01_ was observed during both the H_2_ release and absorption processes.

#### 4.2.2. NiAlH_4_

NiAlH_4_ is also a promising complex hydride that can facilitate the dehydrogenation/hydrogenation process. It has been observed to exhibit low operating temperatures and high hydrogen storage capacity [56]. Compared to other complex borohydrides, alanates, and amides, NiAlH_4_ demonstrates impressive gravimetric and volumetric hydrogen densities of 7.5 wt% and 94 g L^−1^, respectively [57]. The H_2_ storage performance of NiAlH_4_ can be further enhanced by incorporating Ti-based catalysts, which help to reduce the operating temperature and lower the activation energies during H_2_ absorption/desorption process, while maintaining high cyclic stability [58,59]. In this study, we have investigated the role of Ti metal-doped 2D Ti_3_C_2_ MXene in improving the H_2_ storage performance of NiAlH_4_.

The role of pure 2D Ti_3_C_2_ MXene in enhancing H_2_ storage performance was effectively demonstrated by introducing it into NiAlH_4_ for the first time [60]. The addition of 7 wt% Ti_3_C_2_ significantly lowered the dehydrogenation temperature of NiAlH_4_. Furthermore, the incorporation of Ti_3_C_2_ MXene improved the stability of the dehydrogenation process. Following ball milling and the chemical interaction between NiAlH_4_ and Ti_3_C_2_, the Ti_3_C_2_ was reduced to metallic Ti and Ti^3+^ through the breaking of Ti–C bonds. The non-isothermal dehydrogenation of 7 wt% Ti_3_C_2_-doped NiAlH_4_ began at 100 °C, which was significantly lower than the 190 °C observed for pure NiAlH_4_. The hydrogenation onset temperature was 50 °C, with a H_2_ uptake of 4.9 wt% achieved at 150 °C under non-isothermal conditions. In isothermal dehydrogenation tests at 140 °C for 100 min, a H_2_ release of 4.7 wt% was observed. Additionally, the dehydrogenated sample at 100 bar absorbed 4.6 wt% of H_2_ at 120 °C (NiAlH_4_-0.4 wt% H_2_). Overall, the introduction of 2D Ti_3_C_2_ significantly enhanced the H_2_ storage capacity by achieving 4.8 wt% after 10 cycles, with reduced operating temperatures compared to pure NiAlH_4_.

Ti_3_C_2_(OH_0.8_F_1.2_)_2_ MXene derivatives have also gained attention for enhancing the H_2_ storage capacity of NiAlH_4_, specifically by forming a composite of 90% anatase and 10% rutile TiO_2_ in the flower-shaped (FS) FS-A_0.9_R_0.1_-TiO_2_/C (FS-A_0.9_R_0.1_-TC) for the first time [61]. This study demonstrated that Ti_3_C_2_ MXene-derived carbon-supported TiO_2_ promotes H_2_ desorption at lower temperatures. The TPD results showed that the combination of FS-A_0.9_R_0.1_-TC and NiAlH_4_ reduced the onset temperature to 95 °C compared to the 155 °C required for pure NiAlH_4_. The isothermal dehydrogenation was observed with a H_2_ release of 3.11 wt% in 90 min at 100 °C and 4.6 wt% in 20 min at 140 °C. Additionally, stable TPD curves (over 10 cycles) indicated a dehydrogenation of 4.5 wt% after the second cycle. The presence of abundant anatase TiO_2_ (001), graphene-like carbon, and carbon-doped TiO_2_ enhanced the dehydrogenation performance of NiAlH_4_ at lower temperatures when FS-A_0.9_R_0.1_-TC was added.

In another study, the role of partially derived anatase TiO_2_ (A-TiO_2_) at the interface of Ti_3_C_2_ MXene (MXene/A-TiO_2_) was found to enhance the low-temperature H_2_ storage performance of NiAlH_4_ [62]. Different weight percentages of MXene/A-TiO_2_ (0, 5, 10, and 15 wt%) were used as additives to NiAlH_4_ during the ball milling process. TPD graphs revealed that the onset dehydrogenation temperature of NaAlH_4_ + 15 wt% MXene/A-TiO_2_ decreased to 80 °C compared to the 155 °C for pure NiAlH_4_ as shown in Figure 5c. However, the dehydrogenation capacity of 4.21 wt% was achieved, which was lower than the 5.34 wt% observed for NaAlH_4_. The isothermal dehydrogenation process of NaAlH_4_ + 10 wt% MXene/A-TiO_2_ showed a rapid 3 wt% H_2_ release within 7 min, reaching 4.8 wt% after 200 min at 140 °C (Figure 5d). The activation energies for NaAlH_4_ + 15 wt% MXene/A-TiO_2_ were 78.32 and 65.31 kJ mol^−1^ for the first and second dehydrogenation steps, respectively. It should be noted that the porous structure NaAlH_4_ + 10 wt% MXene/A-TiO_2_ facilitated the diffusion and transport of H_2_. Overall, the homogeneous distribution of Ti and C in NiAlH_4_ promoted the Ti-H and TiC formation, which enhanced the H_2_ storage performance.

Jiang et al. [63] demonstrated the enhanced low operating temperature, reduced activation energy, and improved cyclic stability of 2D Ti_3_C_2_ MXene-doped NiAlH_4_. After the ball milling process with Ti_3_C_2_, the NiAlH_4_ phase remained intact, indicating a strong interaction between NaH, Al, and H_2_. In this study, Ti_3_C_2_-doped NaAlH_4_ (NaAlH_4_-8 wt% Ti_3_C_2_) and Ti_3_C_2_-doped NaH/Al (NaH/Al-8 wt% Ti_3_C_2_) were prepared by the ball milling technique to evaluate H_2_ storage performance. The non-isothermal dehydrogenation temperature of hydrided NaH/Al-8 wt% Ti_3_C_2_ was 76 °C, which was lower than that of pure NaAlH_4_ (146 °C). Under isothermal conditions, the hydrided NaH/Al-8 wt% Ti_3_C_2_ released 4.1 wt% H_2_ in 119 min at 110 °C (NaAlH_4_-8 wt% Ti_3_C_2_-174 min). Furthermore, the dehydrogenated NaH/Al-8 wt% Ti_3_C_2_ absorbed 4.2 wt% H_2_ in just 4.5 min at 110 °C. The authors emphasized the importance of the high surface area of 2D Ti_3_C_2_ MXene in enhancing H_2_ absorption performance. The NaH/Al-Ti_3_C_2_ composite exhibited a more distinct and stronger valence state of Ti (Ti^3+^) than Ti^0^. The distribution of Ti and TiF_x_ particles on NaAlH_4_ facilitated rapid H_2_ absorption and release. The above features revealed the activation energies of NaH/Al-8 wt% Ti_3_C_2_ were 92.5 ± 4.6 kJ mol^−1^ (first step) and 58.1 ± 2.9 kJ mol^−1^ (second step).

In another study, the formation of Ti-F-Ce bonds at the interface of 10 wt% CeF_3_/Ti_3_C_2_ was found to influence the hydrogenation and dehydrogenation behavior of NaAlH_4_ [64]. Interestingly, the CeF_3_/Ti_3_C_2_ interface facilitated improved low-temperature H_2_ absorption and release. The observed Ti-F-Ce bonding remained stable after H_2_ absorption and desorption, enhancing the H_2_ storage performance of NaAlH_4_. Additionally, the chemical state of Ti (Ti_0_) in the NaAlH_4_ + 10CeF_3_/Ti_3_C_2_ composite was more stable compared to NaAlH_4_ + 10Ti_3_C_2_ due to the formation of the Ti-F-Ce structure. As a result, the NaAlH_4_ + 10CeF_3_/Ti_3_C_2_ composite promoted the stability of Ti^0^ species, which improved catalytic activity by preventing the formation of Ti-based alloys. With the stability of Ti^0^ species confirmed, non-isothermal dehydrogenation of 4.95 wt% H_2_ was achieved at an initial temperature of 87 °C, significantly lower than the 155 °C for pure NaAlH_4_. Under isothermal conditions, 3.0 wt% H_2_ was released in 80 min, reaching 3.9 wt% after 6 h at 100 °C. Overall, the dehydrogenation process at 100 °C demonstrated practical potential for mobile vehicle applications.

In another study, ball milling of carbon-coated anatase TiO_2_ nanoparticles interfaced with 2D Ti_3_C_2_ (10 wt% C@TiO_2_/Ti_3_C_2_) and NaAlH_4_ resulted in the formation of Ti valence states such as Ti^0^ and Ti^3+^, which were stabilized by suppressing Ti-O and Ti-C bonds for enhancing the H_2_ storage performance at low temperatures [65]. Here, the combination of Ti-containing TiO_2_, Ti_3_C_2_, and carbon proved to be highly effective additives for improving the H_2_ storage performance of NaAlH_4_. Specifically, Ti^0^ and Ti^3+^ played a crucial role in H_2_ desorption, while the carbon in C@TiO_2_/Ti_3_C_2_ significantly contributed to the dehydrogenation process. The presence of 2D Ti_3_C_2_ and TiO_2_ effectively lowered both the dehydrogenation temperature and improved hydrogen storage capacity, respectively. For example, the dehydrogenation temperature of NaAlH_4_ + 10 wt% Ti_3_C_2_ was reduced to 85 °C compared to the NaAlH_4_ (155 °C) under non-isothermal conditions. Isothermal studies showed that approximately 4 wt% of H_2_ was released in 13 min at 140 °C (4.91 wt% after 200 min). Furthermore, the activation energies of dehydrogenation were 72.41 kJ mol^−1^ (first step) and 64.27 kJ mol^−1^ (second step), which were lower than the values reported previously for NaH/Al-8 wt% Ti_3_C_2_ (92.5 ± 4.6 kJ mol_-1_ for first step) [63]. Thus, the catalytic effect of C@TiO_2_/Ti_3_C_2_ significantly influenced the H_2_ storage performance of NaAlH_4_.

The potential of Ti^0^ and Ti^3+^ in enhancing H_2_ storage in NaAlH_4_ was further emphasized by the development of N-doped carbon-coated Ti_3_C_2_ (Ti_3_C_2_/NC) [66]. The strategic interaction between pyridinic-N and Ti^0^ significantly improved the H_2_ storage performance. The Ti_3_C_2_/NC composite (10 wt%), mixed with NaAlH_4_, accelerated the non-isothermal dehydrogenation at lower temperatures with onset temperatures of 85 °C, 126 °C (first step), and 168 °C (second step), releasing 4.84 wt% H_2_, which was lower than the NaAlH_4_ dehydrogenation temperature (155 °C). The onset temperature of 85 °C was slightly lower than that observed in CeF_3_/Ti_3_C_2_ (87 °C). Under isothermal conditions (140 °C), the Ti_3_C_2_/NC + NaAlH_4_ mixture released H_2_ in amounts of 3.0, 4.0, and 4.61 wt% over 4, 16, and 60 min, respectively. Furthermore, the composite exhibited remarkable dehydrogenation cyclic stability, retaining 96.3% of its capacity (4.66 wt%) after 15 cycles. The stable H_2_ absorption and desorption characteristics were attributed to the formation of Ti^0^ and Ti^3+^ during ball milling. The electron transfer between Ti^0^ and pyridinic-N, as well as from pyridinic-N to Ti^0^, was responsible for the release of H_2_ and absorption, respectively.

#### 4.2.3. LiBH_4_

Lithium borohydride (LiBH_4_) has been explored as a promising lightweight material for H_2_ storage, offering an ultrahigh volumetric density of 121 kg H_2_ m^−3^ and gravimetric density of 18.4 wt% [67]. However, the dehydrogenation/hydrogenation process occurred at high temperatures (>400 °C) due to the strong chemical bonding in LiBH_4_ [68]. As a result, significant efforts have been made to lower the dehydrogenation temperature and improve reversibility [69]. The inclusion of 2D Ti_3_C_2_ has shown to play a crucial role in enhancing the H_2_ storage capacity of LiBH_4_. Although research on Ti_3_C_2_-based LiBH_4_ is still in its early stages, the following studies highlight the potential of Ti_3_C_2_ to improve the H_2_ storage performance of LiBH_4_.

The merit of reversibility of the H_2_ storage process was demonstrated by developing the LiBH_4_@Ti_3_C_2_ composite [70]. Unlike the ball milling methods mentioned earlier, the LiBH_4_@2Ti_3_C_2_ composite was prepared using a simple impregnation method at room temperature, with varying mass ratios (2:1, 1:1, 1:2, and 1:3). Nanosized LiBH_4_ particles were dispersed on and between the Ti_3_C_2_ MXene layers. The TPD curves revealed that the LiBH_4_@2Ti_3_C_2_ (1:2) composite reduced the H_2_ desorption temperature to 172.6 °C, compared to 220 °C for pure LiBH_4_. The isothermal dehydrogenation behavior of LiBH_4_@2Ti_3_C_2_ was 8.2, 11.3, and 12.6 wt% after 8 h at 300, 350, and 380 °C, respectively, with 9.6 wt% H_2_ release at 380 °C within 1 h. However, the dehydrogenation cyclic stability of LiBH_4_@2Ti_3_C_2_ declined from 10.6 wt% in the first cycle to 5.5 wt% in the third cycle after 6 h at 350 °C, showing a 48% decrease after three cycles. This loss in cyclic stability was attributed to the unavoidable agglomeration of LiBH_4_ particles on Ti_3_C_2_, which reached about 100 nm in size, adversely affecting the performance of LiBH_4_@2Ti_3_C_2_.

**Figure 6:**
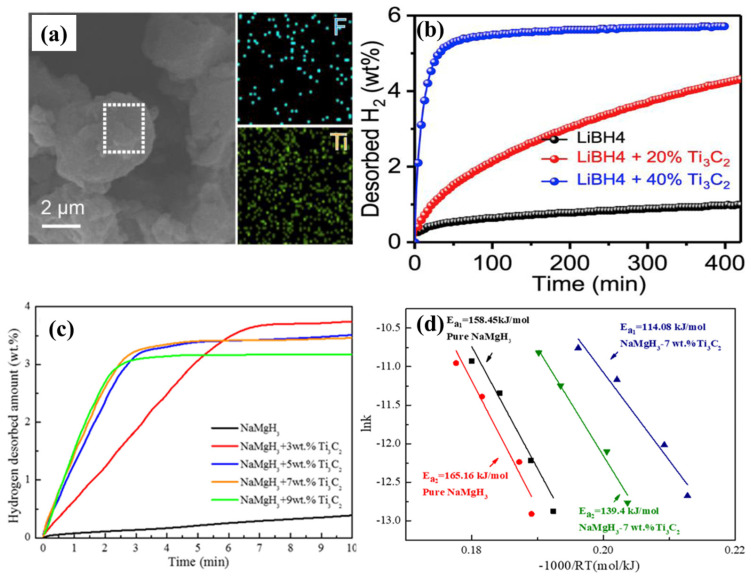
(**a**) Surface morphology and elemental mapping of LiBH_4_ + 40 wt% Ti_3_C_2,_ (**b**) isothermal H_2_ desorption of pure LiBH_4_, LiBH_4_ + 20 wt% Ti_3_C_2_, and LiBH_4_ + 40 wt% Ti_3_C_2_, (reprinted from Ref. [71] copyright 2019, with permission from Elsevier), (**c**) isothermal dehydrogenation behavior of pure NaMgH_3_ and NaMgH_3_-Ti_3_C_2_ at 3, 5, 7, and 9 wt% of Ti_3_C_2_, (**d**) first step of dehydrogenation activation energy of NaMgH_3_ and NaMgH_3_-7 wt%Ti_3_C_2_ (reprinted from Ref. [72] copyright 2021, with permission from Elsevier).

In another study, ball milling of LiBH_4_ with various amounts of Ti_3_C_2_ (x = 0, 20, and 40 wt%) was investigated to enhance the H_2_ storage performance of LiBH_4_ in the LiBH_4_ + x Ti_3_C_2_ [71]. The TPD studies showed a significant reduction in the onset temperature from 300 °C for pure LiBH_4_ to 120 °C for LiBH_4_ with 40 wt% Ti_3_C_2_. The SEM image and elemental mapping of the LiBH_4_ + 40 wt% Ti_3_C_2_ are shown in Figure 6a. Furthermore, isothermal dehydrogenation of LiBH_4_ + 40 wt% Ti_3_C_2_ at 300 °C resulted in a H_2_ release of 3 wt% over 6 h compared to 0.5 wt% for pure LiBH_4_. At 350 °C, the H_2_ release reached 5.37 wt% in 1 h, as shown in Figure 6b. The activation energy for dehydrogenation was also significantly reduced to 70.3 kJ mol⁻¹ for LiBH_4_ + 40 wt% Ti_3_C_2_, compared to pure LiBH_4_ (187 ± 24 kJ mol^−1^). Among various additives (40 wt% TiC, TiO_2_, and Ti_3_C_2_), Ti_3_C_2_ showed the most dominant effect on the dehydrogenation process, exhibiting the lowest onset temperature. This was attributed to the unique properties of Ti_3_C_2_, including its layered structure and carbon sheets in its 2D form. The improved dehydrogenation at lower temperatures was explained by the substitution of H by F and the formation of TiB_2_.

#### 4.2.4. NaMgH_3_

Doping of NaMgH_3_ with 7 wt% Ti_3_C_2_ significantly lowered the dehydrogenation temperature compared to pure NaMgH_3_ [72]. The Ti_3_C_2_ MXene at 7 wt% facilitated a rapid dehydrogenation process (TPD) at 350 °C than other concentrations (3, 5, and 9 wt%). However, higher Ti_3_C_2_ content in the NaMgH_3_-Ti_3_C_2_ composite led to the formation of MgO, which resulted in a decrease in H_2_ desorption efficiency due to the excess “dead weight” of Ti_3_C_2_. The isothermal dehydrogenation of the 7 wt% Ti_3_C_2_ composites released 3.4 wt% H_2_ at 350 °C within 5 min (compared to NaMgH_3_-0.2 wt%) as shown in Figure 6c. The activation energy for the first dehydrogenation step decreased to 114.08 kJ mol-1 for NaMgH_3_ with 7 wt% Ti_3_C_2_, compared to 158.45 kJ mol^−1^ for pure NaMgH_3_ (Figure 6d). In terms of isothermal absorption, NaMgH_3_-7 wt% Ti_3_C_2_ absorbed 3.5 wt% H_2_ at 300 °C in just 6 s, while pure NaMgH_3_ absorbed only 3.0 wt% H_2_ in 15 min. Furthermore, the cyclic stability of NaMgH_3_ improved significantly with the addition of 7 wt% Ti_3_C_2_, increasing from poor stability after five cycles for pure NaMgH_3_. Overall, Ti_3_C_2_ played a crucial role in enhancing the H_2_ storage performance of NaMgH_3_.

Overall, the 2D Ti_3_C_2_ material significantly boosted the H_2_ storage performance in Mg(BH_4_)_2_, NiAlH_4_, LiBH_4_, and NaMgH_3_. The systematic H_2_ storage performance of Mg(BH_4_)_2_, NiAlH_4_, LiBH_4_, and NaMgH_3_ with Ti_3_C_2_ additives is summarized in Table 2.

## 5. Complex Metal Hydrides

In addition to the previously discussed binary and ternary metal hydride combinations for H_2_ storage performance, the inclusion of 2D Ti_3_C_2_ played a significant role in enhancing the performance of complex metal hydrides by reducing the activation energy and operating temperature. The following quaternary metal hydride combinations also demonstrated promising H_2_ storage performance with the incorporation of 2D Ti_3_C_2_.

### 5.1. LiNa_2_AlH_6_ and Li_1.3_Na_1.7_AlH_6_

The addition of 5 wt% Ti_3_C_2_ to LiNa_2_AlH_6_ reduced the dehydrogenation temperature by approximately 68 K compared to pure LiNa_2_AlH_6_ [73]. The Ti^0^ species were actively involved during the dehydrogenation process, where they transformed into Ti^3+^. During the absorption process, Ti^3+^ further converted to Ti^2+^. Notably, the carbon in Ti_3_C_2_ did not interact with H in LiNa_2_AlH_6_. A gradual increase in Ti_3_C_2_ content (up to 5 wt%) effectively lowered the dehydrogenation temperature to 385 K, compared to the higher temperature of 453 K in pure LiNa_2_AlH_6_. The addition of Ti_3_C_2_ induced thermal destabilization of LiNa_2_AlH_6_, which contributed to the lowering of dehydrogenation temperature. This process resulted in improved dehydrogenation and reduction in the activation energy of LiNa_2_AlH_6_. The hydrogenation activation energy for LiNa_2_AlH_6_ with 5 wt% Ti_3_C_2_ was 58.28 kJ mol^−1^, which was slightly lower than that of pure LiNa_2_AlH_6_ (63.19 kJ mol^−1^). However, the dehydrogenation activation energy for LiNa_2_AlH_6_ with 5 wt% Ti_3_C_2_ (191 kJ mol^−1^) was higher than that of pure LiNa_2_AlH_6_ (163 kJ mol^−1^). Overall, the hydrogen absorption kinetics performance was superior to the dehydrogenation process.

**Figure 7:**
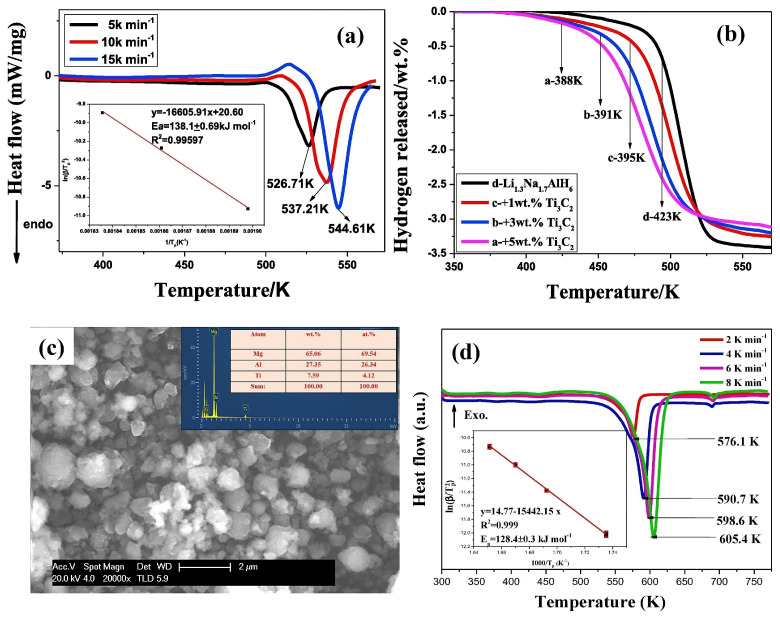
(**a**) Dehydrogenation activation energy of Li_1.3_Na_1.7_AlH_6_ + 5 wt% Ti_3_C_2,_ (**b**) variation in the desorption temperature of Li_1.3_Na_1.7_AlH_6_ and Li_1.3_Na_1.7_AlH_6_ + Ti_3_C_2_ at various wt% (1, 3, and 5) of Ti_3_C_2_ (reprinted from Ref. [74] copyright 2018, with permission from Elsevier), (**c**) surface morphology of 4MgH_2_-LiAlH_4_-Ti_3_C_2_, and (**d**) dehydrogenation activation energy of 4MgH_2_-LiAlH_4_-Ti_3_C_2_ (reprinted from Ref. [75] copyright 2019, with permission from Elsevier).

Fan et al. [74] investigated the active role of 2D Ti_3_C_2_ (1 wt%, 3 wt%, and 5 wt%) in enhancing the H_2_ storage performance of Li_1.3_Na_1.7_AlH_6_. The dehydrogenation activation energy of Li_1.3_Na_1.7_AlH_6_ with 5 wt% Ti_3_C_2_ increased to 231.9 kJ mol^−1^ compared to 138.1 kJ mol^−1^ for pure Li_1.3_Na_1.7_AlH_6_ (Figure 7a). This increase suggests a negative impact on the dehydrogenation kinetics of Li_1.3_Na_1.7_AlH_6_ + 5 wt% Ti_3_C_2_. However, the desorption temperature was slightly reduced in the Li_1.3_Na_1.7_AlH_6_ + 5 wt% Ti_3_C_2_ (388 K) compared to pure Li_1.3_Na_1.7_AlH_6_ (423 K), as shown in Figure 7b. On the other hand, no significant difference was observed in the H_2_ absorption activation energies of Li_1.3_Na_1.7_AlH_6_ + 5 wt% Ti_3_C_2_ (56.3 kJ mol^−1^) compared to pure Li_1.3_Na_1.7_AlH_6_ (59.8 kJ mol^−1^). After the hydrogenation process, both Ti^2+^ and Ti^0^ demonstrated their importance for H_2_ absorption. Overall, Ti_3_C_2_ played a crucial role in improving the H_2_ storage performance of complex metal hydrides.

### 5.2. MgH_2_-LiAlH_4_

The incorporation of the MgH_2_-LiAlH_4_ complex hydride with Ti_3_C_2_ significantly enhanced the de/re-hydrogenation kinetics by promoting the development of metallic Ti and C [75]. The surface morphology of the 4MgH_2_-LiAlH_4_-Ti_3_C_2_ system is shown in Figure 7c. The dehydrogenation temperature of 4MgH_2_-LiAlH_4_-Ti_3_C_2_ reduced to 336 K compared to as-milled 4MgH_2_ (400 K) and 4MgH_2_-LiAlH_4_ (610 K). The system achieved a hydrogen release of 6.6 wt% from 4MgH_2_-LiAlH_4_-Ti_3_C_2_. After dehydrogenation, the Ti^0^ and Ti-O, through the disappearance of Ti-C, revealed the transformation of Ti_3_C_2_ into metallic Ti, which interacted with MgH_2_ for the formation of TiH_1.942_ (Ti^2+^). Here, the dehydrogenation activation energy of 4MgH_2_-LiAlH_4_-Ti_3_C_2_ was 128.4 kJ mol^−1^, lower than that of 4MgH_2_-LiAlH4-176.2 kJ mol^−1^ as shown in Figure 7d. The re-hydrogenation activation energy of 4MgH_2_-LiAlH_4_-Ti_3_C_2_ (65.7 kJ mol^−1^) was also lower than that of 4MgH_2_-LiAlH_4_ (99.2 kJ mol^−1^) with a H_2_ absorption of 3.5 wt% at 582 K in 1000 s under 4 MPa pressure. Overall, the addition of 2D Ti_3_C_2_ played a pivotal role in enhancing the H_2_ storage performance of the 4MgH_2_-LiAlH_4_-Ti_3_C_2_ system.

In conclusion, the 2D Ti_3_C_2_ significantly improved the H_2_ storage performance of LiNa_2_AlH_6_, Li_1.3_Na_1.7_AlH_6_, and 4MgH_2_-LiAlH_4_. The H_2_ storage performance of these systems with the addition of 2D Ti_3_C_2_ MXene is summarized in Table 3.

## 6. Challenges and Future Perspectives

Despite the promising potential of 2D Ti_3_C_2_ MXenes for H_2_ storage, several factors still need further investigation. To date, only a limited number of studies have focused on Ti_3_C_2_ MXene-based metal hydrides. Therefore, the following perspectives are essential to advance the H_2_ storage performance of 2D Ti_3_C_2_ MXene-based metal hydrides:

(i) The role of physisorption or chemisorption of Ti_3_C_2_ MXenes is still challenging. An in-depth discussion on the chemisorption versus physisorption of Ti_3_C_2_ MXenes is needed to improve the H_2_ storage capacity of metal hydrides.
(ii) Surface termination groups provide the room for active sites for hydrogen molecules. The role of surface termination groups in the H_2_ absorption/desorption process of Ti_3_C_2_ MXenes remains insufficiently explored. The role of -OH and -O terminated Ti_3_C_2_ MXene should be studied to strengthen the H_2_ storage performance of metal hydride.
(iii) The precise involvement of Ti_3_C_2_ MXene content in the H_2_ storage process at the interface of metal hydrides is not well understood. Specifically, the roles of edge sites, interlayer spacing, and surface terminations should be addressed.
(iv) A detailed comparison between monolayer, few-layer, and multilayer Ti_3_C_2_ MXenes is necessary to enhance the H_2_ storage process through the variation in the surface area, binding strength, storage and release ability. The direction of morphological tuning has the ability to strengthen the H_2_ absorption and release kinetics during the H_2_ storage process.
(v) Expansion of interlayer spacing and surface area of Ti_3_C_2_ MXene by the doping of single metal atoms have a high chance of accommodating the hydrogen molecules between the layers. These features provide a chance to enhance the H_2_ absorption/desorption processes at the interface of metal hydrides.
(vi) Metal oxides such as SnO_2_, WO_3_, MnO_2_, MoO_3_, and ZrO_2_ gained their importance in H_2_ storage [76]. Thus, the integration of metal oxides with Ti_3_C_2_ MXene has potential room for further improvement in H_2_ storage of various metal hydrides.

The integration of Ti_3_C_2_ MXenes into metal hydrides demonstrates considerable promise for achieving low-temperature adaptable H_2_ storage performance. Thus, the role of 2D Ti_3_C_2_ MXenes as catalysts is crucial for realizing stable and efficient H_2_ storage capabilities.

## 7. Conclusions

The potential of 2D Ti_3_C_2_ MXenes in enhancing the H_2_ storage performance of various metal hydrides has been systematically investigated. Among all the metal hydrides, doping Ti_3_C_2_ into MgH_2_ resulted in remarkable H_2_ storage performance at lower temperatures and in a significantly shorter time. The presence of Ti metal and the various valence states of Ti greatly enhanced the H_2_ storage capabilities. Specifically, the incorporation of Ti_3_C_2_ MXene reduced both the operating and onset temperatures of the metal hydrides. Additionally, the exposure of a few layers and internal edge planes contributed to an increase in active sites, further boosting the H_2_ storage performance. Sharp responses in isothermal dehydrogenation were observed with 6.2 wt% released in 60 s [37], 6.7 wt% in 3 min [43], and 7.0 wt% in 3 min for the addition of pure Ti_3_C_2_, Ti_3_C_2_ MXene-derived K_2_Ti_6_O_13_, and PrF_3_/Ti_3_C_2_ into MgH_2_, respectively. The inclusion of 2D Ti_3_C_2_ MXenes in various metal hydrides also demonstrated enhanced cyclic de/re-hydrogenation stability with 10 cycles for 5 wt% Ni_30_/FL-Ti_3_C_2_T_x_ doped MgH_2_ [39], 10 cycles for Ti_3_C_2_ MXene derived K_2_Ti_6_O_13_ + MgH_2_ [43], 20 cycles for MgH_2_ + Ti_3_C_2_@MnO_2_ [49], 15 cycles for Ti_3_C_2_/NC + NaAlH_4_ [66], and 5 cycles for NaMgH_3_-7 wt% Ti_3_C_2_. These findings strongly emphasize the crucial role of the 2D layered structure of Ti_3_C_2_ MXenes involved in improving the H_2_ storage performance of various metal hydrides, highlighting their potential applicability in future fuel cell technologies.

## Figures and Tables

**Table 1:** Hydrogen storage performance of binary metal hydrides (MgH_2_ and AlH_3_) integrated Ti_3_C_2_ MXenes at a selective weight percentage of Ti_3_C_2_.

Additive (Selective Content)	Isothermal Dehydrogenation	Isothermal Hydrogenation	Activation Energy (kJ mol^−1^) Absorption/Desorption	Key Parameters For H_2_ Storage	Ref.
	Temperature—H_2_ Proportion—Time	Temperature—H_2_ Proportion—Time			
*MgH_2_*					
Ti_3_C_2_ (5 wt%)	300 °C—6.2 wt%—60 s	150 °C—6.1 wt%—30 s	---/98.9 (MgH_2_-155)	Metallic Ti formation	[37]
Ti_3_C_2_/TiO_2_-C (5 wt%)	250 °C—5.0 wt%—1700 s	125 °C—4.0 wt%—800 s	42.32 (MgH_2_-71)/77.69	Anatase TiO_2_, Ti^0^, Ti^2+^ Ti^3+^, and Ti^4+^	[38]
Ni/Ti_3_C_2_T_x_ (5 wt%)	250 °C—5.83 wt%—1800 s	100 °C—5.0 wt%—1700 s	41.36 (MgH_2_-71)/96.36	Ti^0^, Ti^2+^ Ti^3+^, and Ti^4+^	[39]
Ni@Ti_3_C_2_	250 °C—5.2 wt%—15 min	125 °C—5.4 wt%—25 s	73 ± 3.5 (MgH_2_-141 ± 5.3)/56 ± 4	Metallic Ti along with Ni	[41]
Ti_3_C_2_ MXene derived K_2_Ti_6_O_13_ (5 wt%)	280 °C—6.7 wt%—3 min	200 °C—6.5 wt%—30 s	---/105.67 (MgH_2_-175.34)	Ti and Ti^2+^	[44]
2V_2_C/Ti_3_C_2_ (10 wt%)	225 °C—5.1 wt%—60 min	40 °C—5.1 wt%—20 s	---/79.4 (MgH_2_-127.7)	Uniform distribution of Mg, Ti, and V	[45]
Ti_3_C_2_ (6 wt%)	240 °C—6.45 wt%—10 min	150 °C—6.47 wt%—480 s	---/99 (MgH_2_-153)	Metallic Ti formation	[46]
Ni/Ti_3_C_2_ (5 wt%)	100 °C—4.59 wt%—1200 s	250 °C—5.87 wt%—2400 s	42.38 (MgH_2_-71)/91.64	Ti valence states (Ti^0^, Ti^2+^ Ti^3+^, and Ti^4+^)	[47]
PrF_3_/Ti_3_C_2_ (5 wt%)	260 °C—7.0 wt%—3 min	150 °C—6.16 wt%—10 min	---/78.11(MgH_2_-117.98)	Ti valence states (Ti^0^, Ti^2+^, Ti^3+^, and Ti^4+^)	[40]
Ti_3_C_2_ (5 wt%)	---	275 °C—6.6 wt%---	36.22/79.46	Internal metallic Ti active edge sites	[48]
Ti_3_C_2_@MnO_2_	275 °C—6.4 wt%—484 s	30 °C—4.4 wt%—150 s	---/61.8 ± 2.2 (MgH_2_-142.4 ± 0.9)	Multiple interfaces, Ti_3_C_2_/MgH_2_, TiO_2_/MgH_2_, MnO_2_/MgH_2_, MnO/MgH_2_, and Mn/MgH_2_	[50]
Ni/Ti_3_C_2_ (Ti_3_C_2_ @5 wt%)	300 °C—3.96 wt%---	300 °C—4.46 wt%---	---/75.0(Mg-135.4)	Synergy between Ni and Ti_3_C_2_	[51]
Ti_3_C_2_-Ni	523 K—4.982 wt%—60 min	453 K—5.72 wt%—1 min	80.54/---	Mg-TiCrV/Ti_3_C_2_-Ni interface	[49]
*AlH_3_*					
Ti_3_C_2_ (4 wt%)	100 °C—6.9 wt%—20 min	---	---/40	High surface area and active sites of Ti_3_C_2_	[54]

**Table 2:** Hydrogen storage performance of ternary metal hydrides- (Mg(BH_4_)_2_, NiAlH_4_, and LiBH_4_) integrated Ti_3_C_2_ MXenes at an optimized weight percentage of Ti_3_C_2_.

Additive (Content)	Isothermal Dehydrogenation	Isothermal Hydrogenation	Activation Energy (kJ mol^−1^) Absorption/Desorption	Key Parameters for H_2_ Storage	Ref.
	Temperature—H_2_ Proportion—Time	Temperature—H_2_ Proportion—Time			
*Mg(BH_4_)_2_*					
VF_4_@Ti_3_C_2_ (20 wt%)	275 °C—8.2 wt%—300 min	---	---/172.9 (Mg(BH_4_)_2_-374.1)	Formation of metallic Ti and VH_2.01_	[55]
*NiAlH_4_*					
Ti_3_C_2_ (7 wt%)	140 °C—4.7 wt%—100 min	120 °C—4.6 wt%—60 min	---/87.3 ± 6.7 (First step)	Ti metal and Ti^3+^	[60]
Ti_3_C_2_ (OH_0.8_F_1.2_)_2_	100 °C—3.11 wt%—90 min	---	---	Ti_3_C_2_ MXene-derived alanates and rutile TiO_2_	[61]
MXene/A-TiO_2_ (15 wt%)	140 °C—3.0 wt%—7 min	---	---/78.32 (First step)	Homogeneous distribution of Ti and C	[62]
Ti_3_C_2_ (8 wt%)	110 °C—4.1 wt%—119 min	110 °C—4.2 wt%—4.5 min	---/92.5 (First step)	Ti and TiF_x_ particles	[63]
CeF_3_/Ti_3_C_2_	100 °C—3.0 wt%—80 min	---	---/81.39 (First step)	Ti-F-Ce bonding	[64]
C@TiO_2_/Ti_3_C_2_ (10 wt%)	140 °C—4.0 wt%—13 min	---	---/72.41 (First step)	Ti^0^ and Ti^3+^ states	[65]
Ti_3_C_2_/N doped carbon (10 wt%)	140 °C—4.61 wt%—60 min	---	---/76.66 (First step)	Interaction between pyridinic-N and Ti^0^	[66]
*LiBH_4_*					
Ti_3_C_2_	300 °C—8.2 wt%—8 h	---	---/94.44 (50% of LiBH_4_)	Ti-containing defect sites	[70]
Ti_3_C_2_ (40 wt%)	300 °C—3.0 wt%—6 h	---	---/70.3 (LiBH_4_-187 ± 24)	Ti metal and high surface area	[71]
*NaMgH_3_*					
Ti_3_C_2_ (7 wt%)	350 °C—3.4 wt%—5 min	300 °C—3.5 wt%—6 s	---/114.08 (NaMgH_3_-158.45) (First step)	Lamellar-structure Ti_3_C_2_	[72]

**Table 3:** Complex metal hydrides (LiNa_2_AlH_6_, Li_1.3_Na_1.7_AlH_6_, and 4MgH_2_-LiAlH_4_) integrated Ti_3_C_2_ MXenes for H_2_ storage performance.

Complex Metal Hydrides						Key Parameters for H_2_ Storage	Ref.
Additive	Dehydrogenation Temperature		Hydrogenation Activation Energy (kJ mol^−1^)		H_2_ Release/ Absorption (wt%)		
Ti_3_C_2_	LiNa_2_AlH_6_ + 5 wt% Ti_3_C_2_ (385 K)	LiNa_2_AlH_6_ (453 K)	LiNa_2_AlH_6_ + 5 wt% Ti_3_C_2_ (58.28)	LiNa_2_AlH_6_ (63.19)	---	Ti^0^ species	[73]
Ti_3_C_2_	Li_1.3_Na_1.7_AlH_6_ + 5 wt% Ti_3_C_2_ (388 K)	Li_1.3_Na_1.7_AlH_6_ (423 K)	Li_1.3_Na_1.7_AlH_6_ + 5 wt% Ti_3_C_2_ (56.3)	Li_1.3_Na_1.7_AlH_6_ (59.8)	---	Ti^2+^ and Ti^0^	[74]
Ti_3_C_2_	4MgH_2_-LiAlH_4_-Ti_3_C_2_ (400 K)	4MgH_2_-LiAlH_4_ (610 K)	4MgH_2_-LiAlH_4_-Ti_3_C_2_ (65.7)	4MgH_2_-LiAlH_4_ (99.2)	6.6/3.5	metallic Ti, TiH_1.942_ (Ti^2+^)	[75]
